# Drug-induced hyperglycemia in obstetrics: key offending drugs, mechanisms, and practical management

**DOI:** 10.3389/fphar.2026.1866528

**Published:** 2026-09-17

**Authors:** Jingyi Xu, Thu Nguyet Nguyen, Tianwen Liu, Fengjun Jiang, Xiying Zhao, Shumin Qin, Jinjin Yin

**Affiliations:** 1 The Second Clinical College of Guangzhou University of Chinese Medicine, Guangzhou, China; 2 Department of Gastroenterology, the Second Affiliated Hospital of Guangzhou University of Chinese Medicine, Guangdong Provincial Key Laboratory of Clinical Research on Traditional Chinese Medicine Syndrome, Guangzhou, China; 3 Guangdong Provincial Key Laboratory of Clinical Research on Traditional Chinese Medicine Syndrome, Guangzhou, China; 4 Department of Pharmacy, Guangdong Provincial Key Laboratory of Major Obstetric Diseases, Guangdong Provincial Clinical Research Center for Obstetrics and Gynecology, the Third Affiliated Hospital, Guangzhou Medical University, Guangzhou, China; 5 State Key Laboratory of Traditional Chinese Medicine Syndrome, Guangdong Key Laboratory for Translational Cancer Research of Chinese Medicine, International Institute for Translational Chinese Medicine, Guangzhou University of Chinese Medicine, Guangzhou, Guangdong, China

**Keywords:** blood glucose management, drug-induced hyperglycemia, mechanism, gestational diabetes, obstetrics

## Abstract

**Introduction:**

Drug-induced hyperglycemia is an important metabolic complication that may arise when medications disrupt insulin secretion, insulin sensitivity, or glucose homeostasis. In obstetric practice, several commonly used drugs, including glucocorticoids, progesterone, antihypertensive agents, tocolytic drugs, and immunosuppressants, may impair glycemic control to varying degrees, and prolonged exposure may even lead to drug-induced diabetes mellitus. These effects are particularly important in pregnant women with pregestational diabetes, gestational diabetes, obesity, or other risk factors for dysglycemia.

**Methods:**

We systematically reviewed the literature from major databases over the past decade on the association between commonly used obstetric medications and hyperglycemia, with a focus on glucocorticoids, beta-adrenergic agonists, tacrolimus, nifedipine, and indomethacin. Evidence from clinical studies, case reports, and mechanistic investigations was summarized.

**Results:**

Among the drugs reviewed, glucocorticoids, beta-adrenergic agonists, and tacrolimus showed relatively prominent diabetogenic effects, mainly through increased insulin resistance, β-cell dysfunction, and reduced insulin secretion. In contrast, nifedipine and indomethacin appeared to have less clinically significant effects on blood glucose. The use of high-risk agents during pregnancy was associated with a higher incidence of hyperglycemia and increased complexity of glycemic management.

**Discussion:**

Early identification of high-risk drugs and high-risk patients, together with individualized glucose monitoring and timely intervention, may reduce related complications and improve medication safety during pregnancy. Further prospective studies are warranted to define safe thresholds across different gestational stages and maternal conditions.

## 1 Introduction

Certain drugs can interfere with insulin synthesis, secretion, and action, thereby resulting in drug-induced hyperglycemia. Although its precise prevalence remains unclear, increasing numbers of cases of drug-induced hyperglycemia have been reported in recent years (Jain and Lai, 2024). Drug classes commonly associated with hyperglycemia include hormonal agents, antihypertensive drugs, lipid-lowering agents, anti-asthmatic drugs, antipsychotics, antiretroviral agents, antibacterial drugs, antitumor agents, and immunosuppressants (Fathallah et al., 2015; Heurtebize and Faillie, 2024). In obstetric practice, several commonly used medications, including glucocorticoids for promoting fetal lung maturation, progesterone, antihypertensive drugs, tocolytic agents, and immunosuppressants, may elevate blood glucose levels to varying extents, and prolonged use of some of these agents may even result in drug-induced diabetes mellitus (Jain and Lai, 2024). Such hyperglycemia can increase the burden of inpatient management and may contribute to adverse pregnancy outcomes, including preterm delivery (Wang et al., 2025b; Burt et al., 2026). This review summarizes current evidence regarding hyperglycemia and diabetes mellitus associated with drugs commonly used in obstetrics, with the aim of facilitating early identification of high-risk medications, improving understanding of the underlying mechanisms, and guiding preventive strategies to reduce the incidence of drug-induced hyperglycemia and its related complications, thereby enhancing medication safety in pregnant women. (Figure 1). This narrative review was based on a targeted literature search of PubMed, Embase, Web of Science, and Google Scholar for articles published primarily from January 2000 to March 2025, with earlier landmark studies included when considered clinically or mechanistically important. Search terms included combinations of “drug-induced hyperglycemia,” “drug-induced diabetes,” “pregnancy,” “obstetrics,” “gestational diabetes,” “glucocorticoids,” “progesterone,” “dydrogesterone,” “17-hydroxyprogesterone caproate,” “thyroid hormone,” “antihypertensive drugs,” “beta-blockers,” “tocolytics,” “immunosuppressants,” “tacrolimus,” and “cyclosporine.” We primarily selected English-language articles that were most relevant to obstetric practice, including review articles, clinical trials, cohort studies, case-control studies, practice guidelines, and key mechanistic studies. Reference lists of important articles were also screened to identify additional relevant publications. As this was a narrative review, the literature search and article selection were not conducted according to a formal systematic-review protocol.

**FIGURE 1:**
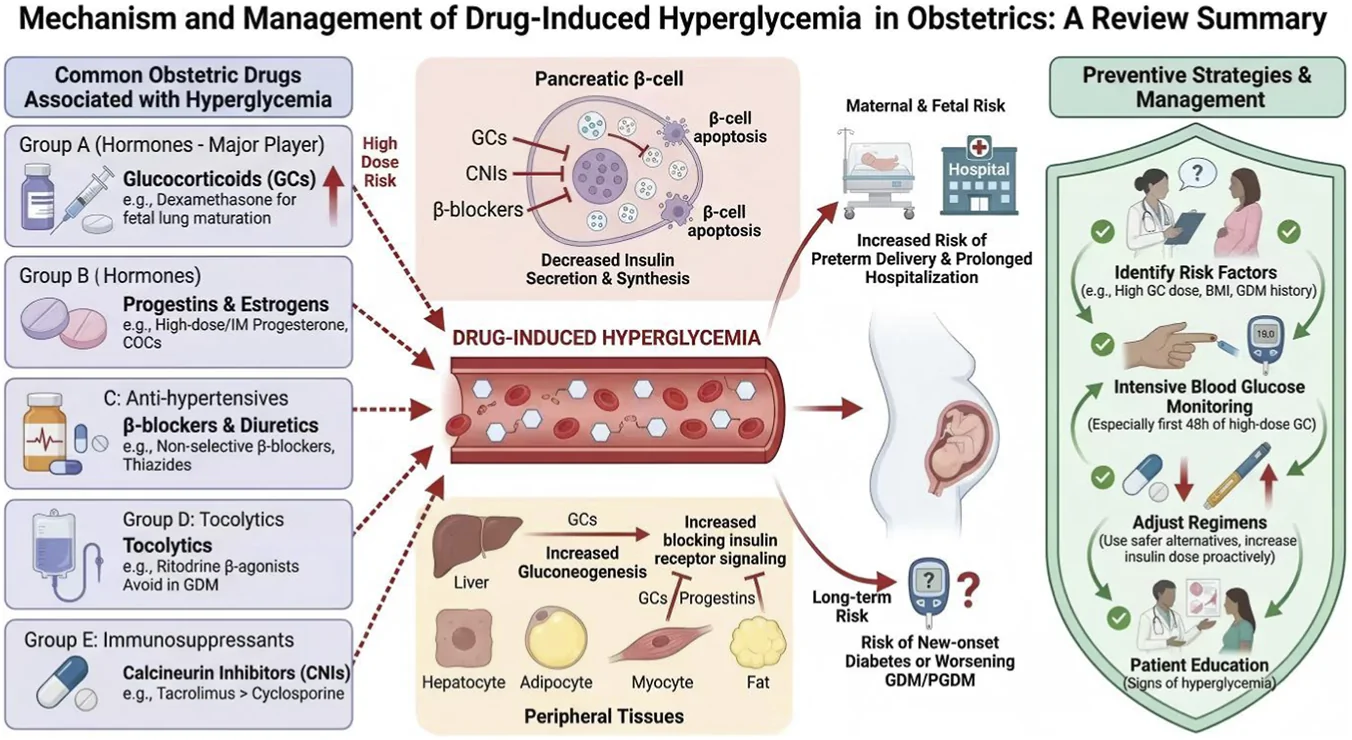
Overview of drug‐induced hyperglycemia in obstetrics: mechanisms, risks, and management. This schematic summarizes the key aspects of drug‐induced hyperglycemia in obstetric care. Common implicated medications include glucocorticoids (the highest‐risk class, e.g., dexamethasone for fetal lung maturation), progestins/estrogens, antihypertensives, tocolytics, and immunosuppressants. The primary pathophysiological mechanisms are pancreatic β‐cell dysfunction (apoptosis and reduced insulin synthesis/secretion) and peripheral insulin resistance (enhanced hepatic gluconeogenesis and impaired insulin signaling in peripheral tissues). Associated risks include short‐term maternal‐fetal complications (preterm delivery, prolonged hospitalization) and long‐term metabolic risks (new‐onset or worsening diabetes). Core management strategies involve risk factor identification, intensive glucose monitoring (especially during high‐dose glucocorticoid therapy), regimen adjustment, and patient education.

## 2 Drugs associated with hyperglycemia

### 2.1 Hormonal agents

#### 2.1.1 Glucocorticoid

Glucocorticoids (GCs) are widely used in obstetrics for conditions such as rheumatic immune disorders, recurrent miscarriage, thrombocytopenia during pregnancy, and promotion of fetal lung maturation. One of their major adverse effects is hyperglycemia, including new-onset hyperglycemia in patients without a history of diabetes mellitus and severe uncontrolled hyperglycemia in those with pre-existing diabetes mellitus (Suh and Park, 2017). A recent meta-analysis reported that the prevalences of GC-induced diabetes and hyperglycemia were 18.6% and 32.3%, respectively (Liu et al., 2014). One study estimated that 64% of hospitalized patients receiving high-dose GCs (≥40 mg/day of prednisone or an equivalent dose of another GC) would develop hyperglycemia (Donihi et al., 2006). GCs may induce hyperglycemia regardless of the route of administration (oral, injectable, or inhaled), provided that the administered dose exceeds the physiological range (Gulliford et al., 2006). The underlying mechanisms of GC-induced hyperglycemia are complex and include increased insulin resistance, enhanced gluconeogenesis, and reduced insulin secretion (Nakamura et al., 2021). Studies have shown that several factors are strongly associated with GC-induced hyperglycemia, including higher steroid doses (prednisolone >20 mg, hydrocortisone >50 mg, or dexamethasone >4 mg), longer duration of steroid therapy, advanced age, higher body mass index, pre-existing glucose intolerance or impaired glucose tolerance, a personal history of gestational diabetes mellitus or prior GC-induced hyperglycemia, a family history of diabetes mellitus, and HbA1c ≥ 6% (Suh and Park, 2017). Because hyperglycemia and its associated complications in hospitalized patients are significantly associated with prolonged hospitalization, careful monitoring and management are particularly important in patients receiving glucocorticoids, especially at high doses. When an intermediate-acting GC is administered once daily in the morning, its hyperglycemic effect generally becomes evident approximately 4 h after dosing, peaks at around 8 h, and subsides after 12–16 h. In contrast, the hyperglycemic effect of dexamethasone appears about 4 h after administration and may persist for 24–36 h (Wallace and Metzger, 2018). In summary, for patients receiving high-dose GCs, blood glucose should be monitored for at least 48 h, and insulin therapy should be initiated when appropriate. Early adjustment of insulin dosage is recommended for pregnant women already receiving insulin therapy (Wright et al., 2025). The latest Diabetes UK guidelines recommend that, in pregnant women receiving GCs to promote fetal lung maturation, basal and prandial insulin doses may be proactively increased by 50%–80% to prevent GC-induced hyperglycemia (Dashora et al., 2022). The 2018 Canadian Clinical Practice Guidelines provide a standardized protocol for insulin dose adjustment during the 7 days following the first dose of betamethasone: on day 1, increase the basal insulin dose by 25%; on days 2 and 3, increase all insulin doses by 40%; on day 4, increase all insulin doses by 20%; on day 5, increase all insulin doses by 10%–20%; and on days 6 and 7, gradually reduce insulin doses to the pre-betamethasone levels (Canadian Diabetes Association Clinical Practice Guidelines Expert et al., 2013). It should be emphasized that concern about transient glucocorticoid-induced hyperglycemia should not delay the administration of antenatal corticosteroids when they are clinically indicated for fetal lung maturation. In such cases, the maternal and fetal benefits of timely corticosteroid administration generally outweigh the risk of short-term hyperglycemia. The preferred strategy is not to withhold or postpone treatment, but to implement intensified glucose monitoring and timely adjustment of insulin or other glucose-lowering measures when necessary.

#### 2.1.2 Estrogen and progestins

Previous studies on combined oral contraceptives (COCs) have suggested that high-dose formulations may increase the risk of abnormal glucose metabolism, particularly by reducing carbohydrate tolerance (De Leo et al., 2016). Their metabolic effects appear to result from a combination of ethinylestradiol-induced insulin resistance and progestin-related alterations in insulin kinetics and insulin sensitivity (Sitruk-Ware and Nath, 2011). Progestins with androgenic properties have been associated with a greater reduction in insulin sensitivity than those with antiandrogenic activity (Cagnacci et al., 2009; Paschou and Papanas, 2019). However, a systematic review found that estrogen-containing contraceptives exert only limited effects on glucose metabolism in women without diabetes mellitus, and there is no strong evidence that they increase the risk of diabetes mellitus (Lopez et al., 2019).

In obstetric practice, commonly used progestational agents include natural progesterone, dydrogesterone, and 17α-hydroxyprogesterone caproate (17-OHPC), and these agents should not be considered metabolically equivalent. Progesterone receptors are expressed in pancreatic β-cells, and progesterone has been reported to reduce peripheral insulin sensitivity and affect glucose homeostasis during pregnancy; accordingly, it is often regarded as a potentially diabetogenic steroid (Hill et al., 2021). In women with prior gestational diabetes mellitus, long-term follow-up data have suggested that the use of progestin-only pills may be associated with an increased risk of subsequent type 2 diabetes mellitus (Xiang et al., 2010). Experimental studies have also suggested that high progesterone concentrations may induce islet-cell apoptosis, increase insulin resistance, and elevate blood glucose levels (Nunes et al., 2014). Nevertheless, the metabolic effects of progesterone are likely to vary according to formulation and route of administration. Commonly used formulations such as micronized progesterone and vaginal progesterone gel result in relatively low systemic exposure, as only a limited fraction reaches the circulation (Paulson et al., 2014). In the setting of luteal support after IVF-ET, although serum progesterone levels are lower after vaginal administration than after intramuscular injection, pregnancy and live birth rates do not appear to differ significantly between the two routes (Liu and Wu, 2020). Therefore, when progesterone is used for luteal support or pregnancy maintenance, vaginal formulations may be preferred in women at increased metabolic risk because of their lower systemic exposure.

Dydrogesterone is a synthetic progestin without androgenic activity and appears to have minimal effects on glucose metabolism (Hill et al., 2021). Available evidence suggests that its use during pregnancy does not increase the risk of gestational diabetes mellitus (GDM) (Alizadeh et al., 2022). 17α-hydroxyprogesterone caproate (17-OHPC), is a synthetic progestogen that was previously used for the prevention of recurrent spontaneous preterm birth. However, its clinical role has become increasingly controversial. Although earlier studies suggested potential benefit, subsequent confirmatory trials failed to demonstrate consistent efficacy in reducing recurrent preterm birth. As a result, the regulatory approval of 17-OHPC for this indication has been withdrawn in some countries, including the United States, and its routine use is no longer recommended by several professional bodies. Therefore, when discussing the metabolic effects of 17-OHPC, its limited and changing clinical role should also be considered. With respect to glycemic outcomes, previous studies have reported conflicting findings (Rebarber et al., 2007; Wolfe et al., 2011). A recent meta-analysis found that women with singleton pregnancies who received weekly 17-OHPC had higher glucose challenge test values and a higher incidence of GDM than those who did not receive the drug; however, this association was no longer significant when the analysis was restricted to randomized controlled trials (Eke et al., 2019). These findings suggest that the available evidence is inconclusive and may be influenced by confounding in observational studies.

Taken together, routine glucose monitoring is unlikely to be necessary for most women receiving oral dydrogesterone or vaginal progesterone preparations. However, closer glycemic surveillance may be considered in women receiving intramuscular progesterone or 17-OHPC, particularly in those with pre-existing diabetes mellitus, prior GDM, obesity, or other risk factors for dysglycemia.

#### 2.1.3 Thyroid hormones

Levothyroxine is not generally considered a major diabetogenic drug, and reports of levothyroxine-induced hyperglycemia are uncommon. However, thyroid hormones may influence glucose metabolism, particularly when treatment results in excessive thyroid hormone exposure or when therapy is initiated or adjusted in women with pre-existing diabetes mellitus or gestational diabetes mellitus. Thyroid hormones can affect glucose homeostasis through actions on the gastrointestinal tract, liver, skeletal muscle, adipose tissue, and pancreas. Excess thyroid hormone levels may increase intestinal glucose absorption, enhance hepatic glucose output, alter peripheral glucose utilization, and reduce glycogen storage (Chauhan and Patel, 2024). Therefore, in pregnant women with dysglycemia, blood glucose may need to be monitored after initiation, dose adjustment, or discontinuation of levothyroxine therapy, and glucose-lowering treatment should be adjusted when clinically necessary. Importantly (Biondi et al., 2019), when levothyroxine is clinically indicated for overt hypothyroidism or selected cases of subclinical hypothyroidism during pregnancy, treatment should not be withheld solely because of concerns regarding possible changes in glycemic control (Ostadrahimi et al., 2023).

### 2.2 Anti-hypertension drugs

Commonly used antihypertensive drugs during pregnancy include adrenergic receptor blockers, calcium channel blockers, and centrally acting antiadrenergic agents. The oral antihypertensive agents most commonly used during pregnancy are nifedipine, labetalol, and methyldopa. If blood pressure is not adequately controlled with oral medication, intravenous antihypertensive therapy may be used. Commonly used intravenous agents include phentolamine, nicardipine, nitroglycerin, sodium nitroprusside, and urapidil. In addition, diuretics such as furosemide may be used in selected cases (Wang et al., 2025a). As early as 2007, a network meta-analysis suggested that angiotensin-converting enzyme inhibitors (ACEIs) and angiotensin receptor blockers (ARBs) were associated with the lowest risk of new-onset diabetes mellitus, followed by calcium channel blockers (CCBs), beta-blockers, and thiazide diuretics (Wang et al., 2025a). A meta-analysis published in 2021 similarly showed that ACEIs and ARBs were associated with a reduced risk of new-onset diabetes, whereas thiazide diuretics and beta-blockers increased this risk by approximately 20% and 48%, respectively. In contrast, calcium channel blockers had no significant effect (Nazarzadeh et al., 2021). However, ACEIs and ARBs are contraindicated during pregnancy because of concerns regarding adverse effects on fetal development.

#### 2.2.1 Diuretic

Diuretics are generally not recommended for pregnant women with preeclampsia. Potent diuretics, such as furosemide, may be considered only in cases of generalized edema, pulmonary edema, cerebral edema, renal insufficiency, or acute heart failure (Cifkova, 2023). Findings from a pharmacogenetic study showed that diuretics were significantly associated with an increased risk of new-onset diabetes mellitus (Shen et al., 2013). Thiazide diuretics may increase blood glucose levels. Potential mechanisms include reduced insulin sensitivity and impaired insulin secretion. In addition, these agents may cause hypokalemia, which is generally reversible after drug discontinuation (Prichard et al., 1992; Taylor et al., 2006). Furosemide is generally considered to have a neutral effect on endocrine metabolism (Liu et al., 2023). However, some individual studies have suggested that it may also increase blood glucose levels, although the underlying mechanism remains unclear. When hyperglycemia occurs, it usually develops after 2–4 weeks of treatment and typically manifests as a mild elevation in fasting blood glucose. This effect may be more pronounced in patients with diabetes mellitus. If hypokalemia is present during treatment, it should be corrected promptly. Patients with clinically significant hyperglycemia may require dietary modification or insulin therapy (Gifford, 1976). It has also been suggested that the effect of furosemide on glucose tolerance may vary according to the timing of administration, with evening dosing being associated with greater increases in blood glucose than early morning dosing (Fujimura et al., 1991).

#### 2.2.2 β-blocker

Similar to thiazide diuretics, clinical trials have shown that beta-blockers may increase the risk of hyperglycemia and new-onset diabetes mellitus (Gress et al., 2000). However, results from the 2013 NAVIGATOR study showed that beta-blocker use was not associated with new-onset diabetes, which differed from previous findings and may be attributable to differences among beta-blocker classes (Shen et al., 2013). Beta-blockers can be broadly classified into three generations: first-generation nonselective beta-blockers, such as propranolol; second-generation selective β1-blockers, such as metoprolol; and third-generation beta-blockers with vasodilatory properties, including α/β-blockers such as labetalol and carvedilol (Pedersen and Cockcroft, 2007). Previous studies have suggested that β-receptor selectivity is associated with the degree of inhibition of insulin secretion. The lower a drug’s selectivity for β1-receptors, the greater its inhibitory effect on insulin secretion and the higher its potential to induce hyperglycemia (Wicklmayr et al., 1990). A meta-analysis showed that the use of nonselective beta-blockers was associated with a 22% increased risk of new-onset diabetes (Bangalore et al., 2007). Other studies have categorized beta-blockers into nonvasodilating agents (e.g., metoprolol and propranolol) and vasodilating agents (e.g., labetalol and carvedilol). These studies suggest that vasodilating beta-blockers have less adverse impact on insulin sensitivity and glycemic control, are associated with a lower risk of new-onset diabetes, and may improve glucose and lipid metabolism (Gress et al., 2000; Fonseca, 2010). One possible explanation is that nonvasodilating beta-blockers, such as atenolol, metoprolol, and propranolol, lower blood pressure primarily by reducing myocardial contractility and cardiac output. The resulting decrease in cardiac output may trigger compensatory peripheral vasoconstriction to maintain blood pressure, thereby increasing systemic vascular resistance. Reduced perfusion of peripheral tissues, particularly skeletal muscle, may adversely affect glucose and lipid metabolism, promote endothelial dysfunction, and contribute to the development of diabetes mellitus (Le Brocq et al., 2008). In contrast, vasodilating beta-blockers may increase peripheral blood flow, thereby facilitating glucose delivery to skeletal muscle and improving insulin sensitivity (Deedwania, 2011). Small studies have shown that labetalol may have a neutral or even beneficial effect on glucose metabolism (Ohman et al., 1985). However, it is important to note that although labetalol does not typically cause hyperglycemia, its inhibition of catecholamine-induced glycogenolysis may predispose patients to hypoglycemia (Klarr et al., 1994). In addition, labetalol may mask the symptoms of hypoglycemia, such as sweating and tachycardia, making hypoglycemic episodes more difficult to recognize and potentially leading to serious consequences (Mateu-Salat and Corcoy, 2021). Therefore, careful blood glucose monitoring is required in patients with gestational diabetes mellitus who are receiving this drug.

### 2.3 Tocolytics

Commonly used tocolytic agents include beta-adrenergic receptor agonists, indomethacin, nifedipine, and oxytocin receptor antagonists. Most current studies on the effects of nifedipine on blood glucose have concluded that it does not produce predictable or clinically significant adverse effects on glycemic control (Hong et al., 2024). Hyperglycemia has been reported in fewer than 1% of patients receiving indomethacin in clinical trials, published reports, and post-marketing surveillance. Although indomethacin appears to have little clinically significant effect on maternal blood glucose, its use as a tocolytic should be determined according to gestational age and standard obstetric indications. Because fetal risks, including premature constriction of the ductus arteriosus and oligohydramnios, increase with advancing gestation and longer exposure, indomethacin is generally used for short-term tocolysis in earlier gestation and should be avoided or used with extreme caution at later gestational ages according to current obstetric guidance. According to the prescribing information, absolute contraindications to ritodrine use include maternal heart disease, abnormal liver function, preeclampsia, antepartum hemorrhage, uncontrolled diabetes mellitus, tachycardia, hypokalemia, pulmonary hypertension, hyperthyroidism, and chorioamnionitis. Relative contraindications include diabetes mellitus, migraine, and paroxysmal tachycardia. Therefore, ritodrine should be avoided whenever possible in patients with preterm labor complicated by pregestational diabetes mellitus (PGDM) or gestational diabetes mellitus (GDM). Current studies have shown that ritodrine use in diabetic patients increases insulin requirements. If blood glucose levels are not closely monitored and managed in a timely manner, severe hyperglycemia or even ketoacidosis may occur, particularly with intravenous administration (Ogawa et al., 2013). Therefore, if patients with well-controlled GDM require ritodrine for the management of preterm labor, blood glucose and serum potassium levels should be closely monitored, and insulin doses should be adjusted promptly when necessary to avoid serious adverse events. Atosiban is generally well tolerated, and most maternal adverse effects are mild. However, the prescribing information indicates that it may increase blood glucose levels, with a reported incidence of 1%–10%. Therefore, blood glucose should be closely monitored during atosiban treatment, especially in patients with preterm labor complicated by PGDM or GDM (Hong et al., 2024). In summary, for patients with preterm labor complicated by gestational diabetes mellitus or pregestational diabetes mellitus, nifedipine or indomethacin may be preferred as tocolytic agents.

### 2.4 Calcineurin inhibitors

Cyclosporine and tacrolimus are both calcineurin inhibitors (CNIs), a class of immunosuppressive agents widely used after organ transplantation. During pregnancy, they are most commonly used for the management of autoimmune diseases (Flint et al., 2016). Studies have shown that the incidence of post-transplant hyperglycemia and new-onset diabetes ranges from 15% to 50% and is significantly associated with CNI use (Chakkera and Mandarino, 2013). The mechanisms by which CNIs contribute to glucose dysregulation and diabetes are complex and may involve both insulin resistance and impaired insulin secretion (Chakkera et al., 2017). Both CNIs exert direct inhibitory effects on pancreatic β-cells, thereby reducing insulin secretion. Compared with cyclosporine, tacrolimus causes more severe swelling and vacuolization of pancreatic islet cells (Drachenberg et al., 1999). In addition, several studies have shown that tacrolimus has a more pronounced dose-dependent inhibitory effect on insulin secretion (van Hooff et al., 2004). In patients receiving tacrolimus-based immunosuppression, the incidence of diabetes during the first year is significantly associated with trough concentrations >15 ng/mL (Rodrigo et al., 2005). When the trough concentration remains within the therapeutic reference range, the diabetogenic effect appears to be attenuated, possibly because lower drug exposure allows partial recovery of pancreatic β-cell secretory capacity (Boots et al., 2002). Cyclosporine also has diabetogenic potential, but this effect is substantially weaker than that of tacrolimus (Webster et al., 2005). Therefore, in pregnant patients with hyperglycemia receiving tacrolimus, if glycemic control remains poor despite dose reduction within the therapeutic range, switching from tacrolimus to cyclosporine may be considered (Prokai et al., 2012). Tacrolimus is generally regarded as more diabetogenic than cyclosporine in nonpregnant transplant populations; however, pregnancy-specific comparative evidence is limited. Therefore, in pregnant patients with persistent hyperglycaemia during tacrolimus therapy, treatment decisions should be individualised, with priority given to close glucose monitoring and appropriate glycaemic management. Any decision to switch from tacrolimus to cyclosporine should be made cautiously based on maternal and fetal considerations, rather than relying solely on evidence from nonpregnant populations. Regardless of which CNI is used, close blood glucose monitoring is essential (Table 1).

**TABLE 1:** Clinical management of commonly used obstetric drugs associated with hyperglycemia.

Drug class	Representative agents	Major mechanisms of hyperglycemia	Patients at increased risk	Monitoring priorities	Practical management recommendations	Preferred alternatives or optimization strategies
Glucocorticoids	Betamethasone, dexamethasone, prednisolone, hydrocortisone	Increased insulin resistance, enhanced hepatic gluconeogenesis, and reduced insulin secretion	Women with pregestational diabetes mellitus (PGDM), gestational diabetes mellitus (GDM), obesity, impaired glucose tolerance, prior steroid-induced hyperglycemia, family history of diabetes, or elevated HbA1c	Monitor blood glucose for at least 48 h after administration; particular attention should be paid to post-dose and postprandial excursions; insulin-treated women require early reassessment	Intensify glucose monitoring in all high-dose glucocorticoid users; initiate or escalate insulin therapy when needed; in women already on insulin, proactive dose adjustment should be considered	When glucocorticoids are clinically indispensable, management should focus on anticipatory monitoring and temporary insulin intensification, especially after antenatal corticosteroids for fetal lung maturation
Progestins and progesterone-related agents	Progesterone, dydrogesterone, 17α-hydroxyprogesterone caproate, vaginal progesterone preparations	Reduced peripheral insulin sensitivity and disturbance of glucose homeostasis; high progesterone concentrations may impair pancreatic β-cell function	Women with PGDM, GDM, obesity, prior dysglycemia, or prolonged exposure to intramuscular progesterone formulations	Routine intensified monitoring is usually unnecessary for oral dydrogesterone or vaginal progesterone in low-risk women; closer glucose surveillance is advisable with intramuscular progesterone or 17α-hydroxyprogesterone caproate	Individualize monitoring according to baseline metabolic risk and route of administration; women at high risk should undergo additional fasting and postprandial glucose assessment	When clinically appropriate, vaginal progesterone formulations may be preferred, as systemic exposure is generally lower
Thyroid hormones	Levothyroxine	Thyroid hormone may increase glucose production and alter glucose utilization in peripheral tissues; initiation or dose escalation may worsen glycemic control and increase insulin requirement	Women with GDM or PGDM; those starting, discontinuing, or undergoing dose adjustment of levothyroxine	Closely monitor blood glucose after treatment initiation, dose titration, or drug discontinuation	Continue thyroid replacement when indicated, but promptly adjust glucose-lowering therapy if deterioration in glycemic control occurs	The key strategy is not drug withdrawal, but synchronous glucose monitoring and timely adjustment of antidiabetic treatment
Diuretics	Furosemide; thiazide diuretics (not routinely preferred in pregnancy)	Reduced insulin sensitivity, impaired insulin secretion, and aggravation of hyperglycemia by hypokalemia	Women with diabetes, impaired glucose tolerance, prolonged exposure, or concurrent hypokalemia	Monitor fasting glucose during treatment, especially after 2–4 weeks of use; serum potassium should also be assessed	Correct hypokalemia promptly; consider dietary intervention and insulin therapy when needed; avoid unnecessary long-term use	In pregnancy, diuretics should generally be reserved for clear indications such as pulmonary edema, cerebral edema, cardiac failure, or severe fluid overload
β-blockers	Labetalol, propranolol, metoprolol	Non-vasodilating β-blockers may inhibit insulin secretion and reduce peripheral perfusion, thereby worsening insulin resistance; metabolic effects vary by agent	Women with GDM or PGDM, especially those with labile glycemia or impaired awareness of hypoglycemia	Monitor for both hyperglycemia and hypoglycemia; note that labetalol may mask adrenergic symptoms of hypoglycemia	Blood glucose should be monitored during therapy, especially in women with diabetes; clinicians should remain alert to masked hypoglycemia	When a β-blocker is required in pregnancy, vasodilatory agents such as labetalol are generally preferable, although monitoring remains necessary
Tocolytics	Ritodrine, atosiban, nifedipine, indomethacin	β-adrenergic agonists such as ritodrine can markedly increase blood glucose and insulin requirement; atosiban may also be associated with hyperglycemia; nifedipine has minimal metabolic effects in most reports	Women with PGDM or GDM receiving treatment for threatened preterm labor	During therapy, monitor blood glucose closely; potassium monitoring is also advisable, particularly with ritodrine	Ritodrine should be avoided whenever possible in women with PGDM or GDM; if unavoidable, intensive glucose surveillance and prompt insulin supplementation are required; atosiban also warrants glucose monitoring	In women with PGDM or GDM and threatened preterm labor, nifedipine or indomethacin may be preferred where clinically appropriate
Calcineurin inhibitors	Tacrolimus, cyclosporine	Direct pancreatic β-cell toxicity with reduced insulin secretion, together with worsening insulin resistance; tacrolimus may exert stronger diabetogenic effects	Women with pre-existing dysglycemia, prolonged immunosuppressive exposure, or other metabolic risk factors	Monitor glucose closely after treatment initiation and dose adjustment; persistent hyperglycemia should prompt reassessment	These agents should be regarded as high-risk drugs for dysglycemia; early endocrinology consultation and individualized glucose-lowering therapy may be required	When immunosuppression is necessary, the main strategy is enhanced surveillance and multidisciplinary management, balancing maternal disease control against metabolic risk

Abbreviations: PGDM, pregestational diabetes mellitus; GDM, gestational diabetes mellitus.

This table summarizes practical management recommendations based on currently available evidence and clinical considerations. The intensity of glucose monitoring and therapeutic adjustment should be individualized according to gestational age, baseline glycemic status, drug indication, route of administration, and inpatient or outpatient setting.

## 3 Management

The management of drug-induced hyperglycemia should begin with the identification of risk factors, particularly those that are potentially modifiable. In patients considered to be at high risk of developing hyperglycemia, emphasis should be placed on preventive strategies. When clinically feasible, metabolic status, physical activity, and dietary intake should be optimized before initiating drugs known to increase glucose levels. However, clinically indicated glucocorticoid therapy, especially antenatal corticosteroids administered for fetal benefit, should not be delayed in order to achieve glycaemic control first. Instead, clinicians should anticipate a transient rise in blood glucose after glucocorticoid administration and manage it proactively through intensified glucose monitoring and appropriate treatment, including adjustment or escalation of insulin therapy when needed. In addition, when feasible, clinicians should select agents with a lower risk of hyperglycemia and avoid combining two or more diabetogenic drugs whenever possible. In hospitalized patients with diabetes already receiving insulin therapy, insulin doses may be increased preemptively to offset anticipated drug-induced increases in blood glucose. Finally, standardized protocols for glucose monitoring should be implemented in both hospital and outpatient settings where hyperglycemia-inducing drugs are frequently used. In at-risk populations, monitoring should include patient education regarding the signs and symptoms of hyperglycemia and diabetes, as well as regular blood glucose assessment. The frequency and modality of blood glucose monitoring should be tailored to the specific drug regimen and the patient’s individual risk factors. For example, frequent blood glucose monitoring may be necessary in acutely ill patients receiving high doses of drugs associated with hyperglycemia, such as dexamethasone administered for fetal lung maturation (Figure 2).

**FIGURE 2:**
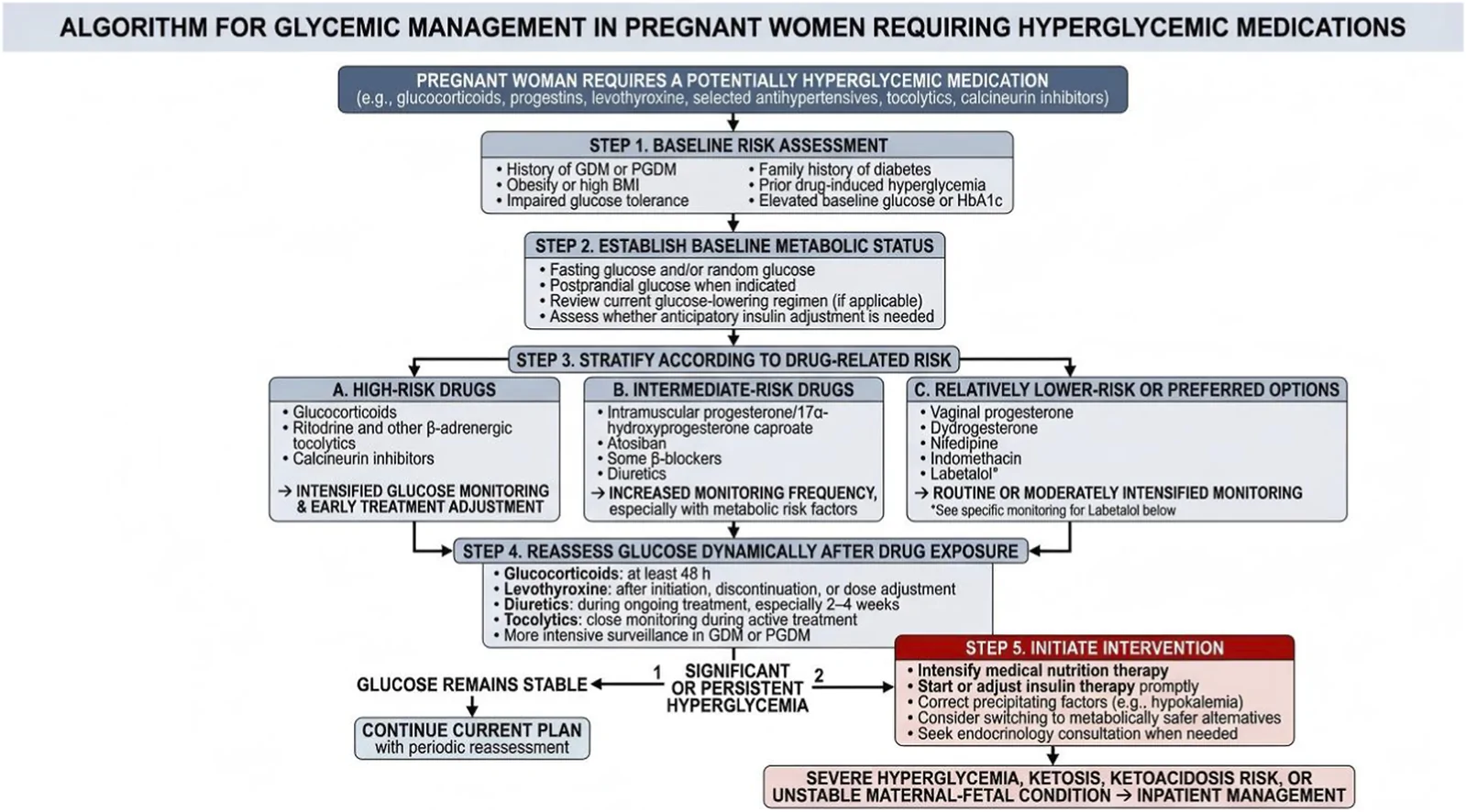
Practical clinical algorithm for the identification and management of drug-induced hyperglycemia in obstetrics. Pregnant women requiring medications with potential hyperglycemic effects should first undergo baseline metabolic risk assessment, including a history of GDM or PGDM, obesity, impaired glucose tolerance, prior drug-induced hyperglycemia, family history of diabetes, and abnormal baseline glucose parameters. After baseline glucose evaluation, patients should be stratified according to the diabetogenic potential of the prescribed drug. High-risk agents, such as glucocorticoids, β-adrenergic tocolytics, and calcineurin inhibitors, require intensified glucose monitoring and early therapeutic adjustment. Intermediate-risk agents warrant closer surveillance in women with underlying metabolic risk, whereas relatively lower-risk agents may be managed with routine or moderately intensified monitoring. If significant or persistent hyperglycemia develops, nutritional intervention, insulin adjustment, correction of precipitating factors, and multidisciplinary consultation should be initiated promptly. Women with severe hyperglycemia, ketosis, or unstable maternalfetal conditions should receive inpatient management.

## 4 Conclusion

Drug-induced hyperglycemia is an important and potentially underrecognized metabolic complication in obstetric practice. Several medications commonly used during pregnancy may impair glycemic control through different mechanisms, including increased insulin resistance, pancreatic β-cell dysfunction, reduced insulin secretion, and altered hepatic glucose metabolism. Among the agents discussed in this review, glucocorticoids, beta-adrenergic agonists, and tacrolimus appear to have relatively greater hyperglycemic potential, whereas nifedipine and indomethacin generally seem to have less clinically significant effects on blood glucose in most settings. These effects are particularly relevant in women with pregestational diabetes mellitus, gestational diabetes mellitus, obesity, or other risk factors for dysglycemia.

From a clinical perspective, early recognition of high-risk drugs and high-risk patients, together with individualized glucose monitoring and timely intervention, may help reduce maternal and neonatal complications and improve medication safety during pregnancy. At the same time, clinically indicated treatment should not be withheld solely because of concerns about transient or manageable drug-related hyperglycemia; rather, careful risk assessment and proactive glycemic management are needed.

However, the currently available evidence remains limited by the predominance of observational studies, heterogeneity in study design and outcome definitions, and the relative lack of robust pregnancy-specific data for several drug classes. Future research should prioritize prospective studies in pregnant populations, clearer quantification of drug-specific glycemic risk, and the development of practical monitoring and management strategies tailored to obstetric care.

## Data Availability

The original contributions presented in the study are included in the article/supplementary material, further inquiries can be directed to the corresponding authors.
